# Effectiveness of platelet-rich fibrin (PRF) as adjunctive therapy in alveolar bone grafting: A systematic review and meta-analysis

**DOI:** 10.1016/j.jobcr.2026.101568

**Published:** 2026-11-03

**Authors:** Husni Mubarak, MuhIrfan Rasul, Nurwahida, Hadira, Yossy Yoanita Ariestiana, Ita Purnama Alwi, A. Tajrin

**Affiliations:** a Department of Oral and Maxillofacial Surgery, Faculty of Dentistry, Hasanuddin University, Makassar, Indonesia; b Hasanuddin University Dental Hospital, Makassar, Indonesia; c Departement of Orthodontic, Faculty of Dentistry, Hasanuddin University, Makassar, Indonesia

**Keywords:** Cleft palate, Alveolar bone grafting, Platelet-rich fibrin, Bone regeneration

## Abstract

**Background:**

Platelet-rich fibrin (PRF) is an autologous biomaterial containing growth factors that may improve bone regeneration and wound healing after alveolar bone grafting (ABG) in patients with cleft-related alveolar defects.

**Objective:**

To evaluate whether PRF used as an adjunct improves the outcome of alveolar bone grafting for alveolar cleft reconstruction.

**Methods:**

We conducted a systematic review and meta-analysis following PRISMA 2020 (PROSPERO: CRD420261447817). PubMed, Embase, and the Cochrane Library were searched up to July 2026. Randomized or non-randomized comparative clinical studies of PRF plus bone grafting versus bone grafting alone were eligible. Outcomes were newly formed bone, graft volume, graft height (resorption), and bone density. Risk of bias was appraised with the Joanna Briggs Institute (JBI) tool and certainty of evidence with GRADE.

**Results:**

Of 1101 records identified, five randomized controlled trials with 126 patients were included. Three trials reported more newly formed bone or less graft resorption with PRF, and two found no difference between groups. In an exploratory meta-analysis of two trials, the random-effects mean difference in newly formed bone was 9.31% (95% CI: −1.07 to 19.70; I^2^ = 83.5%). For graft height (three trials), the mean difference was 0.88 mm (95% CI: −1.13 to 2.90; I^2^ = 89.4%). Bone density, reported by two trials, did not differ between groups. Certainty of evidence was very low for all outcomes.

**Conclusion:**

Current evidence is too limited and too uncertain to show that PRF improves the results of iliac crest bone grafting alone. Larger, well-designed multicenter trials are needed.

## 1 Introduction

Alveolar bone grafting (ABG) is a standard procedure for closing the alveolar cleft in patients with cleft lip and palate.^1^, ^2^ The graft restores alveolar continuity, allows the canine to erupt through bone, stabilizes the maxillary arch, and provides support for later orthodontic and prosthodontic treatment.^2^, ^3^ Recent work has looked at adjuncts that might improve graft healing. One of these is platelet-rich fibrin (PRF), an autologous second-generation platelet concentrate that contains growth factors thought to promote bone and soft tissue healing.^4^, ^5^

Several clinical studies have reported better bone regeneration and faster postoperative healing with PRF.^5^ Others found no significant advantage in bone volume or density over grafting without PRF.^6^, ^7^, ^8^ PRF may also reduce postoperative complications, although the certainty of that evidence varies between reviews.^7^, ^8^ Some authors have combined PRF with bone substitutes to reduce the amount of iliac bone harvested, with acceptable clinical results.^9^

Because the published findings are inconsistent, a systematic review restricted to PRF is warranted. Earlier reviews in this population pooled PRF with platelet-rich plasma without a separate quantitative synthesis for PRF, combined all autologous platelet derivatives used in secondary alveoloplasty, or focused mainly on wound dehiscence.^5^, ^7^, ^8^ The present review differs in three respects (Supplementary Table 2): it examines PRF only, as an adjunct to bone grafting; it uses a search updated to July 2026; and it pools radiographic bone regeneration and graft height outcomes and rates their certainty with GRADE. We therefore aimed to assess whether PRF improves the outcomes of alveolar bone grafting.

## 2 Materials and methods

### 2.1 Study design and reporting standard

This systematic review was conducted and reported according to the Preferred Reporting Items for Systematic Reviews and Meta-Analyses (PRISMA) 2020 statement.^10^ The protocol was registered in PROSPERO (ID: CRD420261447817). Because the review used only data from previously published studies, ethical approval and informed consent were not required.

The review assessed PRF as an adjunctive biomaterial in alveolar bone grafting for patients with cleft-related alveolar defects. The review question was framed with the PICOS framework (Population, Intervention, Comparison, Outcomes, and Study design). Table 1 lists the PICOS elements together with all predefined inclusion and exclusion criteria.

**Table 1:** Review question and eligibility criteria (inclusion and exclusion criteria) structured according to the PICOS framework.

Component	Inclusion criteria	Exclusion criteria
**Population (P)**	Patients with cleft lip and/or palate (unilateral or bilateral alveolar cleft; any age or sex) undergoing alveolar bone grafting for cleft-related alveolar defects	Studies unrelated to alveolar bone grafting in cleft patients (e.g., extraction sockets, ridge augmentation, sinus lift, periodontal defects); animal or in vitro studies
**Intervention (I)**	Platelet-rich fibrin (any preparation protocol or form, e.g., membrane, fragments, or gel) used as an adjunct to the bone graft material during alveolar bone grafting	PRF combined with multiple additional biomaterials without isolated evaluation of the PRF effect; studies without a PRF-treated group
**Comparison (C)**	Bone grafting with the same graft material without platelet-rich fibrin	Studies comparing PRF only with platelet-rich plasma (PRP) or other platelet concentrates without a PRF-free grafting control; studies without a comparison group
**Outcomes (O)**	Radiographic or clinical outcomes related to bone regeneration or graft success: newly formed bone/bone fill percentage, bone volume, bone density, vertical bone height, and bone (graft) resorption	Studies not reporting radiographic or clinical outcomes related to bone regeneration or graft success
**Study design (S)**	Randomized controlled trials, prospective comparative studies, retrospective comparative studies, or controlled clinical studies	Narrative reviews, case reports, conference abstracts, editorials, and letters
**Other criteria**	Articles published in English with full text available	Non-English publications; articles without accessible full text

### 2.2 Search strategy

We searched three electronic databases: PubMed (MEDLINE), Embase, and the Cochrane Library. The search was run on July 11, 2026, and combined controlled vocabulary (MeSH and Emtree terms) with free-text terms for alveolar bone grafting, platelet-rich fibrin, and bone regeneration outcomes. The full search strategy for each database is given in Supplementary Table 1. We also hand-searched the reference lists of the included studies and of related systematic reviews.^5^, ^7^, ^8^

### 2.3 Eligibility criteria

The eligibility criteria were set in the registered protocol before the search was run, and Table 1 summarizes them according to PICOS. Comparative clinical studies in humans were eligible: randomized controlled trials, prospective or retrospective comparative studies, and controlled clinical studies. Studies had to enroll patients with cleft lip and/or palate undergoing alveolar bone grafting, compare PRF added to the bone graft with grafting without PRF, report radiographic or clinical outcomes of bone regeneration or graft success, be published in English, and be available in full text. We excluded animal and in vitro studies; narrative reviews, case reports, conference abstracts, editorials, and letters; studies that compared PRF only with platelet-rich plasma; studies that combined PRF with several other biomaterials without evaluating PRF separately; studies unrelated to alveolar bone grafting in cleft patients; and articles whose full text could not be obtained (Table 1).

Three reviewers (HM, IR, and IPA) screened the records from the electronic and manual searches independently and in a blinded manner, following PRISMA 2020.^10^ Selection took place in three stages. Titles were screened first to remove clearly irrelevant records, then the abstracts of potentially eligible studies were assessed, and finally the full texts were checked against the criteria in Table 1. During selection, each article was judged on its relevance to the review question and on its methods, including possible information bias, how participants were selected, and the quality of the analysis. Disagreements were resolved by discussion until the reviewers reached consensus.

The same three reviewers extracted data independently from the full texts using a standardized form and entered them into a structured table. Extracted items were first author, year of publication, country, study design, sample size, participant age and sex, cleft type, graft material, PRF preparation protocol, comparator, follow-up duration, imaging modality, and funding source when reported. The predefined primary outcomes were bone volume, bone density, bone fill percentage, vertical bone height, and bone resorption. We extracted outcome data at every reported follow-up time point. Missing or unclear information was recorded as not reported (NR), and no assumptions were made about it; only explicitly reported results entered the synthesis. Continuous outcomes were expressed as mean differences (MDs) with 95% confidence intervals (CIs), and dichotomous outcomes, where present, as risk ratios (RRs) with 95% CIs.

Extracted data were checked for completeness and consistency before synthesis. All studies reported continuous outcomes as means and standard deviations, so no conversions were needed. Studies whose summary statistics were incomplete and could not be derived from the published data were kept in the qualitative synthesis but left out of the meta-analysis. Missing data were not imputed.

All included studies were summarized qualitatively. When studies were similar enough in participants, interventions, comparators, and outcome measures, their results were pooled in a meta-analysis, using MDs with 95% CIs for continuous outcomes and RRs with 95% CIs for dichotomous outcomes. We expected clinical and methodological differences between studies and therefore used a random-effects model with the DerSimonian–Laird estimator of between-study variance,^11^ with common-effect estimates reported alongside for comparison. Heterogeneity was tested with Cochran's Q and quantified with I^2^, where values of about 25%, 50%, and 75% were taken to indicate low, moderate, and high heterogeneity.^12^ Analyses were run in R with the meta package^13^ and checked independently in Python (SciPy), and p < 0.05 was considered statistically significant. Because random-effects confidence intervals are too narrow when only a few studies are pooled, we repeated each random-effects analysis with the Hartung–Knapp–Sidik–Jonkman (HKSJ) adjustment.^14^ For graft height, all effects were expressed as PRF minus control, so that positive values favor PRF. Trials that reported change from the immediate postoperative value were combined with a trial that reported only follow-up values with balanced baselines, which is acceptable for mean differences.^15^

Study and participant characteristics, interventions, comparators, follow-up, and outcomes are summarized in evidence tables. Pooled estimates with 95% CIs are shown in forest plots, risk of bias judgments are tabulated, and study selection is shown in a PRISMA 2020 flow diagram.^10^

With so few studies and clear clinical differences between them, subgroup analysis and meta-regression would not have been informative, and neither was performed. For graft height, we ran a sensitivity analysis that excluded the trial by Desai et al. (2024),^16^ in which both arms received bone marrow aspirate concentrate. Publication bias could not be tested with funnel plots or Egger's test because fewer than ten studies contributed to each meta-analysis.

The same reviewers assessed risk of bias independently with the Joanna Briggs Institute (JBI) Critical Appraisal Checklist for Randomized Controlled Trials.^17^ The protocol specified the Risk Of Bias In Non-randomized Studies of Interventions (ROBINS-I) tool for any non-randomized studies, but all five included studies were randomized trials. Disagreements were settled by discussion among the three reviewers until they agreed. Certainty of evidence for each outcome was rated with the Grading of Recommendations Assessment, Development and Evaluation (GRADE) approach.^18^, ^19^

## 3 Results

### 3.1 Study selection process

Fig. 1 shows the selection process in a PRISMA 2020 flow diagram. The searches identified 1101 records: 846 from PubMed, 92 from Embase, 161 from the Cochrane Library, and 2 from hand-searching. After duplicates were removed, 895 records remained for title and abstract screening.

**Fig. 1:**
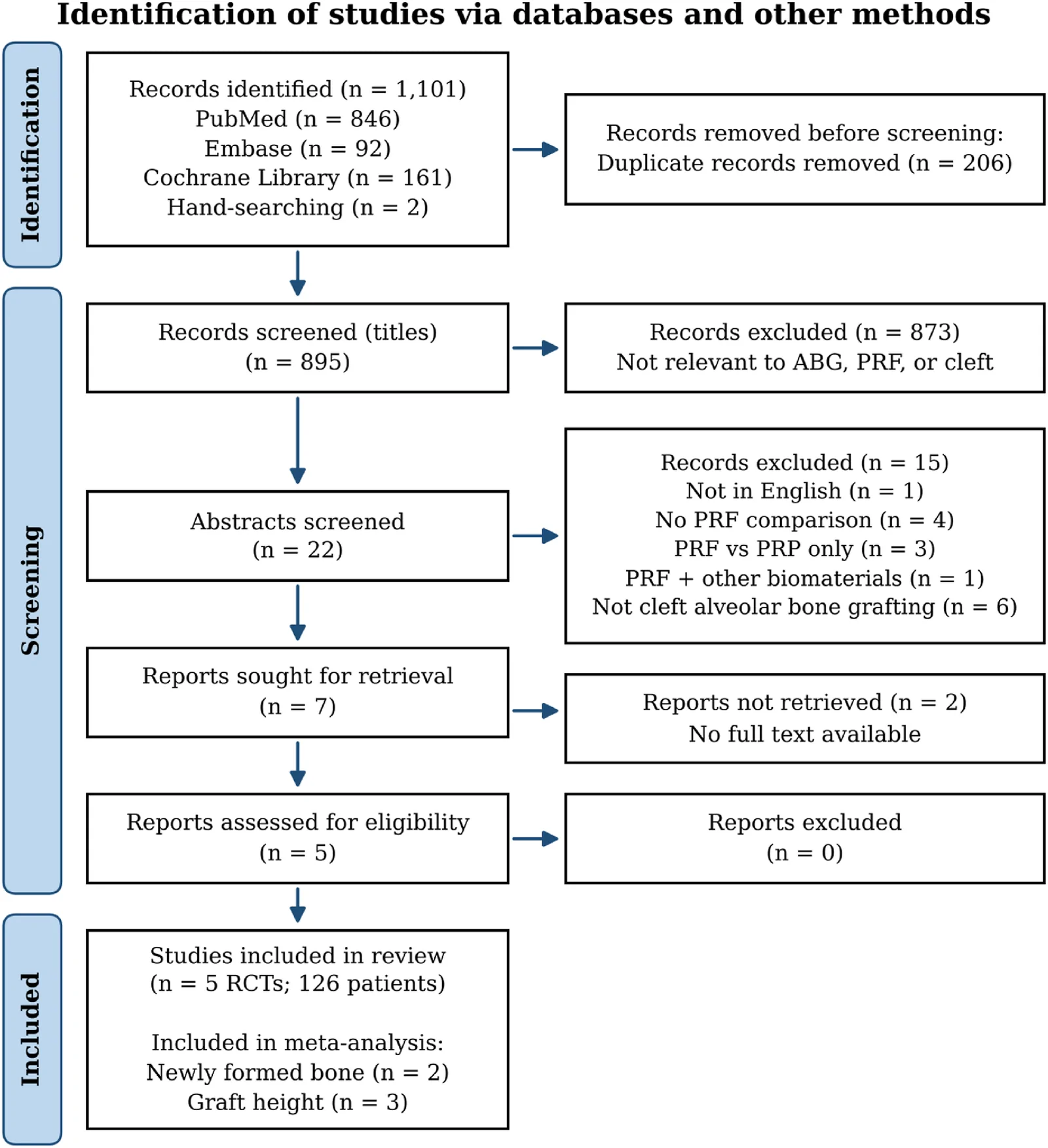
PRISMA 2020 flow diagram of the identification, screening, eligibility assessment, and inclusion of studies evaluating platelet-rich fibrin (PRF) as adjunctive therapy in alveolar bone grafting.

Title screening excluded 873 records that did not concern alveolar bone grafting, PRF, or cleft reconstruction, leaving 22 records for abstract screening. Fifteen of these were excluded: one was not published in English, four had no comparison involving PRF, three compared PRF only with PRP, one combined PRF with other biomaterials without a separate analysis, and six did not concern alveolar cleft bone grafting.

Seven reports were sought for retrieval. Two could not be retrieved in full text despite institutional access and manual searching. The remaining five reports were assessed for eligibility, and all five met the criteria and were included in the review. Two of them contributed to the meta-analysis of newly formed bone and three to the meta-analysis of graft height.

### 3.2 Study and participant characteristics

The five included studies were randomized controlled trials (RCTs) published between 2016 and 2024, with 126 patients in total who underwent secondary alveolar bone grafting. The trials came from four Asian countries: Saudi Arabia (n = 1), Turkey (n = 1), Iran (n = 1), and India (n = 2). Sample sizes ranged from 10 to 40 patients. In the trials that reported age, mean ages ranged from 11.3 to 15.3 years. Three trials enrolled only patients with unilateral clefts, and two included both unilateral and bilateral alveolar clefts (Table 2).^20^, ^21^

**Table 2:** Characteristics of the included studies^16^, ^20^, ^21^, ^22^, ^23^.

No	Author, Years, Geographic Region	Intervention	Comparison	Sample Size	Study Design	Preparation of the PRF	Radiographic Technique	Outcome			
								Newly Formed Bone/Graft Volume		Bone Resorption (graft dimensions)	
								PRF + Iliac	Iliac	PRF + Iliac	Iliac
1	Shawky & Seifeldin, 2016,^22^ Riyadh, Saudi Arabia	PRF + anterior iliac crest bone graft	Iliac Bone Graft	24 (9-14 years; unilateral)	RCT	Venous blood, 3000 rpm for 10 Minutes - Membrane - Cut into small pieces and mixed with cancellous bone	CT Scan (Philips Brilliance 32 Slice Cardiac MDCT), Preoperative and 6 months postoperative	79.74% - 88.4%; (82.6 ± 3.9%)	60.3%–76.4%; (68.38 ± 6.67%)	NR	NR
2	Saruhan & Ertas, 2018^20^ Erzurum, Turkey	Iliac Bone Graft + PRF	Iliac Bone Graft	22 (13 unilateral and 9 bilateral)	RCT	Venous blood, 3000 rpm for 10 min - Membrane	CBCT (Mimics Medical 17.0), Before and 6 months postoperative	50.70% - 80.09% (68.21 ± 10.80%)	47.02% - 79.23% (64.62 ± 9.49%)	NR	NR
3	Omidkhoda et al., 2018^23^ Mashhad, Iran	anterior iliac crest bone + PRF gel	Iliac Bone Graft	10 (9-12 years)	RCT	Venous blood, 3000 rpm for 10 min - PRF was mixed with the bone graft to obtain a substance with a gel-like consistency	CBCT (Planmeca, Finland, 2009). Preoperative and 3 months postoperative	NR	NR	Graft height, mm: Immediate OP (11.3 ± 5.1) 3 months (8.5 ± 3.6)	Graft height, mm: Immediate OP (14.0 ± 4.0) 3 months (10.9 ± 3.6)
4	Desai et al., 2019^21^ Karnataka, India	Iliac Bone Graft + PRF	Iliac Bone Graft	40 (31 unilateral, 9 bilateral)	RCT	Venous blood, 2900 rpm for 10 min - Bone graft chips and PRF clot were mixed with bone particles	CBCT (KODAK 9000C and KODAK 9000C 3D extraoral Imaging System; Carestream Inc., 2016), Radiographic assessment was done preoperatively, immediate postoperatively, and at 3, 6, and 9 months, bone resorption. Type 1—0 to 25% Type 2—25 to 50% Type 3—50to 75% Type 4—75 to 100%.	NR	NR	Graft height, mm: Immediate OP (14.075 ± 1.805) 3 months (13.805 ± 1.857) 6 months (13.210 ± 1.925) 9 months (13.060 ± 1.991)	Graft height, mm: Immediate OP (13.945 ± 1.876) 3 months (13.320 ± 1.842) 6 months (12.50 ± 1.89) 9 months (10.49 ± 1.93)
5	Desai et al., 2024^16^ Karnataka, India	Bone marrow aspirate with PRF along with cancellous iliac bone graft	Bone marrow aspirate concentrate and cancellous Iliac bone graft without PRF	30	RCT	Venous blood, 2900 rpm for 10 min - Membrane	CT Scan, Pre-op, immediate post-op, and 12 months.	Graft volume, cm^3^: Immediate OP (3.02 ± 1.10) 12 months (2.28 ± 0.78)	Graft volume, cm^3^: Immediate OP (2.66 ± 0.54) 12 months (1.95 ± 0.52)	Graft height, mm: Immediate OP (12.63 ± 2.13) 12 months (11.21 ± 1.76)	Graft height, mm: Immediate OP (11.95 ± 1.17) 12 months (10.74 ± 1.37)

Every trial compared iliac crest bone grafting with PRF against iliac bone grafting without PRF. In one trial, both groups also received bone marrow aspirate concentrate with the cancellous iliac graft.^16^ PRF was prepared in a similar way across the trials, from autologous venous blood centrifuged at 2900 to 3000 rpm for 10 min. How it was applied differed: some trials placed PRF as a membrane,^16^, ^20^, ^22^ some cut the membrane into fragments and mixed them with cancellous bone,^21^, ^22^ and one mixed PRF with the graft to form a gel.^23^

Grafts were evaluated with computed tomography (CT) or cone-beam computed tomography (CBCT), and follow-up lasted from 3 to 12 months.^16^, ^20^, ^21^, ^22^, ^23^ The outcomes reported were newly formed bone, graft volume, graft height and width, and bone density.

Three trials measured newly formed bone or graft volume. Shawky and Seifeldin^22^ found more newly formed bone with PRF than without it after six months (82.6 ± 3.9% vs. 68.38 ± 6.67%, a statistically significant difference). Saruhan and Ertas^20^ also reported more bone formation in the PRF group at six months, but the difference was not significant (68.21 ± 10.80% vs. 64.62 ± 9.49%; p > 0.05). In Desai et al. (2024),^16^ the grafted bone volume at 12 months was 2.28 ± 0.78 cm^3^ with PRF and 1.95 ± 0.52 cm^3^ without it (p = 0.19), and the authors reported 75% versus 70% regenerated bone.

Three trials measured graft height over time. In Omidkhoda et al.,^23^ graft height decreased by 2.8 ± 1.7 mm with PRF and 3.1 ± 1.9 mm without it during the first three months, with no significant difference (p = 0.78). In Desai et al. (2019),^21^ graft height at nine months was 13.06 ± 1.99 mm with PRF and 10.49 ± 1.93 mm in the control group, and resorption grades were significantly lower with PRF (p = 0.007). In Desai et al. (2024),^16^ where both groups received bone marrow aspirate concentrate, graft height decreased by 1.42 ± 0.52 mm with PRF and 1.21 ± 0.37 mm without it over 12 months; the authors reported a lower percentage of volumetric resorption with PRF (25% vs. 30%). The results for graft resorption were therefore mixed: one trial favored PRF, and two found no meaningful difference.

Two trials reported bone density, and neither found a significant difference. Shawky and Seifeldin^22^ reported slightly lower density of the newly formed bone with PRF, without statistical significance. Omidkhoda et al.^23^ found that density fell by 101.3 ± 96.1 HU with PRF and 88.7 ± 82.5 HU without it over three months (p = 0.83). Because density was measured with different CT and CBCT scales, the data were not pooled.

### 3.3 Risk of bias assessment

Risk of bias was assessed with the JBI Critical Appraisal Checklist for Randomized Controlled Trials (Table 3).^17^ Two trials were judged to be at moderate risk, two at high risk, and one at moderate-to-high risk.

**Table 3:** JBI critical appraisal of the included randomized controlled trials.^17^.

Question	Shawky & Seifeldin^22^	Saruhan & Ertas^20^	Desai et al. (2019)^21^	Omidkhoda et al.^23^	Desai et al. (2024)^16^
1 Was true randomization used for assignment of participants to treatment groups?	Yes	Unclear	Yes	Unclear	Yes
2 Was allocation to treatment groups concealed?	Unclear	No	Unclear	No	Unclear
3 Were treatment groups similar at the baseline?	Yes	No	Yes	Unclear	Yes
4 Were participants blind to treatment assignment?	No	No	No	No	No
5 Were those delivering treatment blind to treatment assignment?	No	No	No	No	No
6 Were outcomes assessors blind to treatment assignment?	Yes	Yes	Unclear	Unclear	Unclear
7 Were treatment groups treated identically other than the intervention of interest?	Yes	Yes	Yes	Yes	Yes
8 Was follow up complete and if not, were differences between groups in terms of their follow up adequately described and analyzed?	Yes	Yes	Unclear	Yes	Unclear
9 Were participants analyzed in the groups to which they were randomized?	No	No	No	No	No
10 Were outcomes measured in the same way for treatment groups?	Yes	Yes	Yes	Yes	Yes
11 Were outcomes measured in a reliable way?	Yes	Unclear	Unclear	Unclear	Unclear
12 Was appropriate statistical analysis used?	Unclear	Unclear	Unclear	No	Unclear
13 Was the trial design appropriate, and any deviations from the standard RCT design (individual randomization, parallel groups) accounted for in the conduct and analysis of the trial?	Unclear	Unclear	Unclear	Unclear	Unclear

**Overall Risk of Bias**	**Moderate**	**High**	**Moderate**	**High**	**Moderate–High**

The most frequent problems were unclear randomization, inadequate allocation concealment, no blinding of participants or surgeons, no intention-to-treat analysis, and small samples. Performance bias was high in every trial because the surgeon prepares and places the PRF and cannot be blinded to it. Several trials also raised concerns about selection bias, either because the randomization method was poorly described or because the groups differed at baseline.

Detection bias was lower in trials that used CBCT or CT volumetric analysis read by blinded evaluators. Most trials, however, did not report intraobserver or interobserver reliability. Statistical weaknesses were common, including small samples, no power calculation, and incomplete reporting of confidence intervals or of the predefined primary outcome.

Of the five trials, Desai et al. (2019)^21^ had the fewest methodological concerns. The trials by Omidkhoda et al.^23^ and by Saruhan and Ertas^20^ had the most, mainly in selection bias and statistical analysis. With moderate to high risk of bias across the trials, the overall evidence should be read with caution.

### 3.4 Synthesis of results

Only two trials reported newly formed bone, so this meta-analysis is exploratory. Fig. 2 compares PRF plus iliac bone graft with iliac bone graft alone.^20^, ^22^ A positive mean difference (MD) favors the PRF group. In Shawky and Seifeldin's trial,^22^ PRF produced significantly more newly formed bone (MD 14.22%; 95% CI: 9.85 to 18.59). Saruhan and Ertas^20^ also found a difference in favor of PRF, but it was not significant (MD 3.59%; 95% CI: −3.65 to 10.83). The analysis used cleft sites as reported by the trials, so the 22 patients in Saruhan and Ertas^20^ contributed 31 grafted sites.

**Fig. 2:**
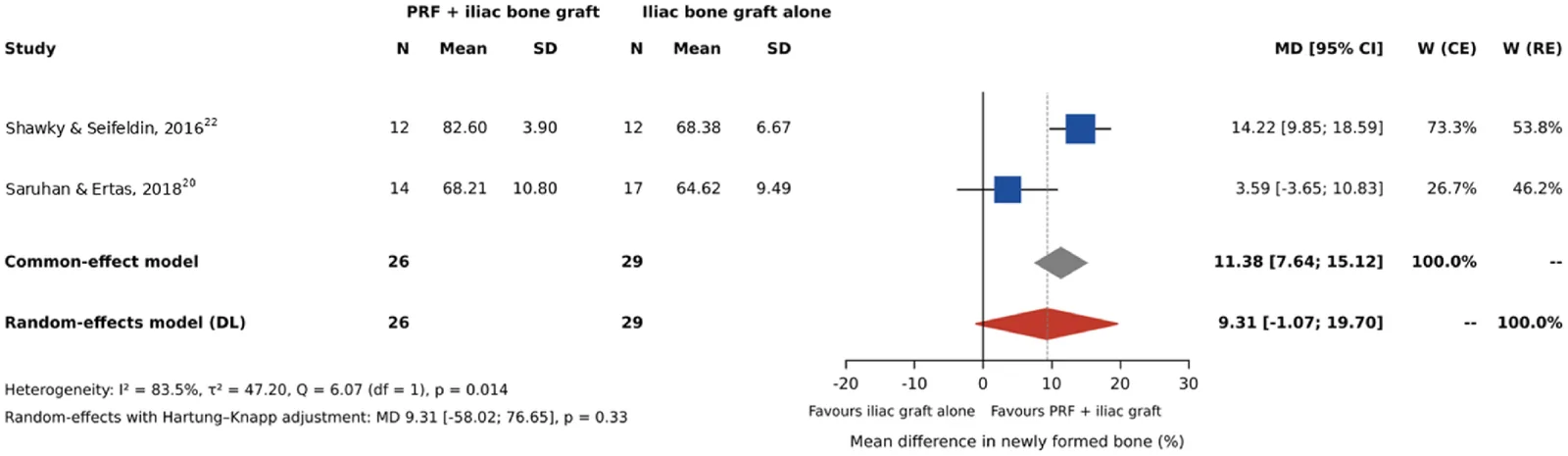
Forest plot of newly formed bone (%) for PRF plus iliac bone graft versus iliac bone graft alone. N, grafted cleft sites; MD, mean difference; CI, confidence interval; W, weight; CE, common-effect model; RE, random-effects model; DL, DerSimonian–Laird estimator.

The common-effect model showed significantly more newly formed bone with PRF (MD 11.38%; 95% CI: 7.64 to 15.12). The random-effects model did not (MD 9.31%; 95% CI: −1.07 to 19.70; p = 0.08). Heterogeneity was high (I^2^ = 83.5%, τ^2^ = 47.20, p = 0.014), so the random-effects estimate is the more appropriate one.^11^, ^12^ With the HKSJ adjustment, the confidence interval widened to −58.02 to 76.65 (p = 0.33), which shows how little information two heterogeneous trials provide. The direction of the pooled effect favors PRF, but the data do not show a statistically significant benefit.

Fig. 3 shows the meta-analysis of graft height, the outcome used to represent graft resorption.^16^, ^21^, ^23^ Positive MDs indicate more preserved graft height with PRF. Omidkhoda et al.^23^ and Desai et al. (2024)^16^ contributed the change from the immediate postoperative value, at 3 and 12 months, respectively, and Desai et al. (2019)^21^ contributed graft height at nine months, when baseline values were similar in both groups (14.08 vs. 13.95 mm).

**Fig. 3:**
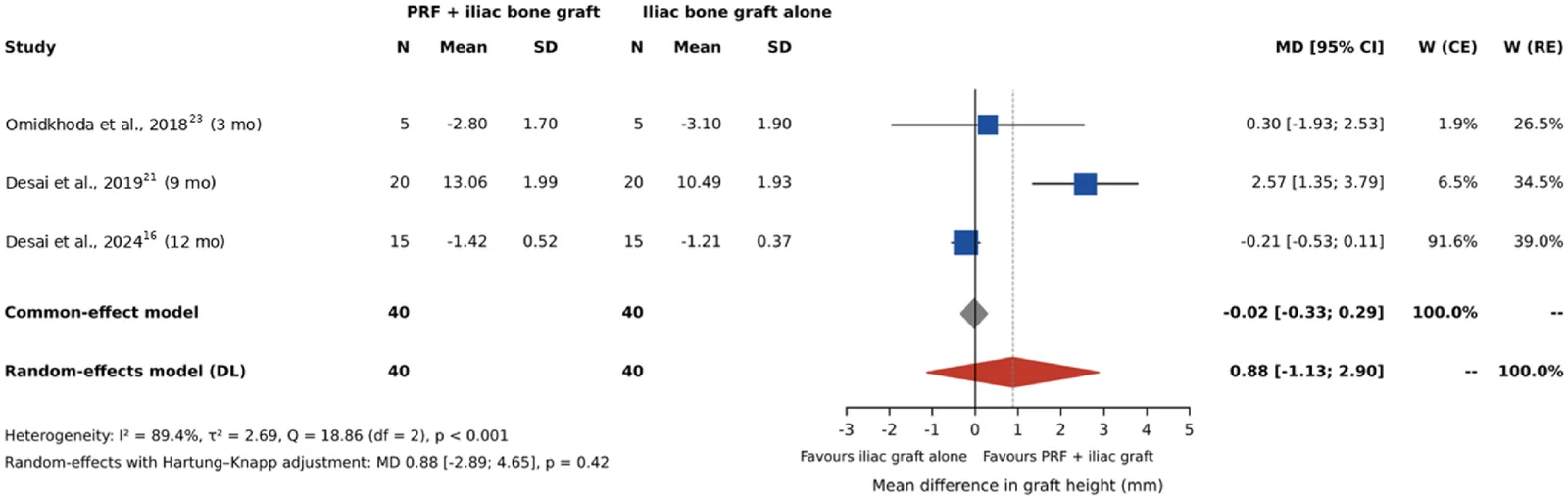
Forest plot of graft height (mm) for PRF plus iliac bone graft versus iliac bone graft alone. Two trials contributed the change from the immediate postoperative value (at 3 and 12 months) and one trial contributed graft height at 9 months; in one trial, both arms received bone marrow aspirate concentrate. Positive values favor PRF. MD, mean difference; CI, confidence interval; W, weight; CE, common-effect model; RE, random-effects model; DL, DerSimonian–Laird estimator.

Only Desai et al. (2019)^21^ showed a significant difference in favor of PRF (MD 2.57 mm; 95% CI: 1.35 to 3.79). The other two trials showed no difference (Omidkhoda et al.,^23^ MD 0.30 mm; 95% CI: −1.93 to 2.53; Desai et al., 2024,^16^ MD −0.21 mm; 95% CI: −0.53 to 0.11). The pooled random-effects MD was 0.88 mm (95% CI: −1.13 to 2.90; p = 0.39), with very high heterogeneity (I^2^ = 89.4%, τ^2^ = 2.69, p < 0.001); the HKSJ interval was −2.89 to 4.65 mm. The common-effect estimate, dominated by the precise Desai et al. (2024)^16^ trial, was −0.02 mm (95% CI: −0.33 to 0.29).

Excluding Desai et al. (2024),^16^ in which both arms received bone marrow aspirate concentrate, gave a random-effects MD of 1.64 mm (95% CI: −0.55 to 3.83; I^2^ = 67.3%; HKSJ 95% CI: −12.56 to 15.83). The sensitivity analysis therefore did not change the conclusion: the available trials do not show that PRF reduces graft resorption.

### 3.5 Certainty of evidence

Certainty of evidence was very low for all outcomes (Table 4). Evidence from randomized trials starts at high certainty in GRADE.^18^, ^19^ It was downgraded for risk of bias, because every outcome included at least one trial judged at high or moderate-to-high risk, and for imprecision, because of the small samples and wide confidence intervals. Newly formed bone and graft height were also downgraded for inconsistency (I^2^ above 80%). Graft volume and bone density came from one and two trials, respectively, and were downgraded twice for imprecision. Very low certainty means that the true effect is likely to differ substantially from the estimates reported here.

**Table 4:** GRADE evidence profile.^18^, ^19^ Comparison: PRF + autogenous iliac bone graft versus autogenous iliac bone graft alone in alveolar bone grafting.

Outcomes	No. Of studies	Study design	Risk of bias	Inconsistency	Indirectness	Imprecision	Publication bias	Overall certainty
Newly formed bone	2 RCTs (46 patients; 55 cleft sites)^20^, ^22^	RCTs	**Serious**^a^	**Serious**^b^	Not serious	**Serious**^c^	Not assessable^d^	**⊕◯◯◯ Very low**
Graft height (resorption)	3 RCTs (80 patients)^16^, ^21^, ^23^	RCTs	**Serious**^a^	**Serious**^b^	Not serious	**Serious**^c^	Not assessable^d^	**⊕◯◯◯ Very low**
Graft volume	1 RCT (30 patients)^16^	RCT	**Serious**^a^	Not applicable	Not serious	**Very serious**^e^	Not assessable^d^	**⊕◯◯◯ Very low**
Bone density (narrative)	2 RCTs (34 patients)^22^, ^23^	RCTs	**Serious**^a^	Not serious	Not serious	**Very serious**^e^	Not assessable^d^	**⊕◯◯◯ Very low**

a At least one contributing trial at high or moderate-to-high risk of bias (Table 3).

b I^2^ above 80%.

c Small samples; 95% CI compatible with no effect and with appreciable benefit.

d Fewer than ten studies; funnel plot and Egger's test not feasible.

e Single trial or very small trials without a pooled estimate. RCT, randomized controlled trial.

## 4 Discussion

This review assessed PRF as an adjunct to alveolar bone grafting (ABG) in patients with cleft-related alveolar defects. Three of the five trials reported more newly formed bone or less graft resorption with PRF, while two found no difference.^16^, ^20^, ^21^, ^22^, ^23^ When the data were pooled, the effects were not statistically significant and the certainty of evidence was very low, so the positive findings of single trials are best treated as hypotheses to test. Earlier reviews reached a similar conclusion: the evidence is heterogeneous and does not show that platelet concentrates are superior to conventional grafting alone.^1^, ^5^, ^8^

The proposed mechanism rests on the composition of PRF, an autologous fibrin matrix that holds platelets, leukocytes, cytokines, and growth factors such as platelet-derived growth factor (PDGF), transforming growth factor beta (TGF-β), and vascular endothelial growth factor (VEGF).^4^, ^24^ These mediators stimulate angiogenesis, osteoblast differentiation, collagen synthesis, and tissue remodeling, all of which are needed for alveolar bone to regenerate.^24^, ^25^ Because the fibrin matrix releases growth factors gradually, PRF may also help early wound healing and graft maturation, although the release pattern depends on the preparation protocol.^26^

The size and direction of the effect varied between trials.^16^, ^20^, ^21^, ^22^, ^23^ Thangarajah et al., reviewing both PRF and platelet-rich plasma, found bone height, density, and volume similar to those achieved with conventional grafting.^5^ Within our review, Desai et al. (2019) found clearly better preservation of graft height with PRF at nine months,^21^ whereas the later trial by the same group, in which both arms also received bone marrow aspirate concentrate, found similar height loss in both groups.^16^ A benefit of PRF on graft stability is therefore possible but unconfirmed. Similar uncertainty surrounds PRF in other bone regenerative procedures.^27^

Much of this variability probably comes from differences in methods between trials.^28^ The trials differed in how PRF was prepared, centrifugation speed, graft material, imaging modality, length of follow-up, and how outcomes were measured.^29^ They may also have used different types of PRF. Leukocyte- and platelet-rich fibrin (L-PRF), advanced PRF (A-PRF), and injectable PRF (i-PRF) differ in their biological properties and in how quickly they release growth factors.^26^, ^30^

Autogenous iliac crest bone remains the reference standard for secondary alveolar bone grafting.^1^, ^31^, ^32^ Current evidence supports PRF as an addition to autogenous bone, not as a substitute for it.^5^, ^8^, ^33^ Cancellous iliac bone is osteogenic, osteoinductive, and osteoconductive, which explains why it remains the most reliable graft material.^2^, ^31^ A recent meta-analysis of alternative biomaterials and combined grafting approaches also found that autogenous grafts gave the most predictable results in cleft reconstruction.^1^

PRF may also improve postoperative recovery. In other oral and maxillofacial procedures, PRF has reduced postoperative pain and swelling and improved soft tissue healing.^25^, ^29^ This could matter in cleft patients, in whom wound dehiscence and graft exposure can cause the graft to fail.^7^, ^8^ Comparable benefits have been reported in guided bone regeneration, alveolar ridge preservation, and the treatment of periodontal intrabony defects.^27^, ^34^, ^35^, ^36^, ^37^.

PRF research in this field lacks standardization. Blood collection, centrifugation settings, clot preparation, and the timing of PRF application were reported inconsistently,^30^ which makes the trials hard to reproduce and may explain some of the conflicting results.^5^ Recent meta-analyses have recommended standardized PRF protocols, three-dimensional radiographic measurement, and longer follow-up in future trials.^28^, ^36^

This review has limitations, some arising from the available evidence and some from our methods. First, we searched only three databases (PubMed/MEDLINE, Embase, and the Cochrane Library), together with reference lists. We did not search Scopus, Web of Science, LILACS, Google Scholar, grey literature, or trial registries such as ClinicalTrials.gov and the WHO ICTRP. Searches that include Web of Science and Google Scholar retrieve more relevant studies,^38^ so we may have missed eligible trials, especially those in regional journals not indexed in our three databases or never published, and publication bias cannot be ruled out. We also limited the search to English-language articles, and one record was excluded for this reason. This may have introduced language bias, although empirical studies suggest that language restrictions rarely change pooled estimates much.^39^ Future updates of this review should search these additional sources.

Second, there were few eligible trials, and most had small samples and short follow-up, which lowers statistical power and makes bias more likely. Several trials also had weaknesses in allocation concealment, blinding, and outcome reporting (Table 3). Third, each meta-analysis combined only two or three trials with different follow-up periods and imaging methods (CT and CBCT), and heterogeneity was high. The gap between the common-effect and random-effects results, and the very wide HKSJ intervals, show that the pooled estimates are not robust. The findings of the individual trials deserve at least as much weight as the pooled numbers.

Fourth, in Desai et al. (2024), both groups received bone marrow aspirate concentrate with the cancellous iliac graft,^16^ which makes this trial less comparable with the others. Excluding it did not change the conclusion for graft height. Fifth, the graft height analysis combined change scores with follow-up values from one trial; this is acceptable for mean differences when baselines are balanced, but it adds a further source of variation. Sixth, two trials included patients with bilateral alveolar clefts,^20^, ^21^ and the reports do not make clear whether grafts or patients were the unit of analysis. If two grafts from the same patient were analyzed as independent observations, the correlation between them was ignored and the pooled estimates may look more precise than they are.

There are still good biological reasons to expect PRF to help bone regeneration. PRF may promote osteogenesis by increasing angiogenesis and modulating inflammation.^24^, ^40^ In laboratory studies, PRF increased osteoblast migration, proliferation, and differentiation and raised the expression of osteogenic markers such as alkaline phosphatase, Runx 2, and osteocalcin.^24^, ^41^

PRF also has practical advantages. It is autologous, cheap, quick to prepare during surgery, and needs no anticoagulants or other additives.^4^, ^24^ This makes it attractive for cleft surgery, particularly in centers with limited resources.^5^ Combining PRF with bone grafts might also reduce donor-site morbidity by improving graft integration and lowering the volume of bone that has to be harvested,^9^, ^33^ but randomized trials in cleft patients have not yet tested this.

## 5 Conclusion

Evidence from five small randomized controlled trials does not show that adjunctive platelet-rich fibrin (PRF) improves the results of autogenous iliac crest bone grafting in secondary alveolar cleft reconstruction. Three trials reported more newly formed bone or less graft resorption with PRF, but the exploratory pooled estimates for newly formed bone and graft height were not statistically significant, heterogeneity was high, and the certainty of evidence was very low. At present, PRF is an inexpensive autologous adjunct with an unproven regenerative benefit in alveolar cleft grafting, not an established standard of care. Multicenter randomized trials with standardized PRF protocols, CBCT volumetric outcomes, the patient as the unit of analysis, and at least 12 months of follow-up are needed before clinical recommendations can be made.

## Statement of informed consent

Because this study is a systematic review and meta-analysis of previously published data and did not involve the recruitment of new human participants, additional informed consent was not required for this review. Informed consent was obtained from all individual participants (or their legal guardians) included in the original studies, as reported by the respective primary investigators.

## Registration and protocol

This systematic review was prospectively registered in the International Prospective Register of Systematic Reviews (PROSPERO) under registration number CRD420261447817. The following changes were made after registration, during peer review: bone resorption was analyzed as the between-group difference in graft height, Hartung–Knapp–Sidik–Jonkman sensitivity analyses were added, and a sensitivity analysis excluding the trial with bone marrow aspirate concentrate in both arms was performed.

## Author contributions

Husni Mubarak: Conceptualization, Methodology, Investigation (screening, data extraction), Writing – original draft, Writing – review & editing.

Irfan Rasul: Investigation (screening, data extraction), Formal analysis, Writing – review & editing.

Ita Purnama Alwi: Investigation (screening, data extraction), Validation, Writing – review & editing.

Andi Tajrin: Validation, Supervision, Formal analysis.

Nurwahida: Validation, Supervision, Formal analysis.

Hadira: Validation, Supervision, Formal analysis.

Yossy Yoanita: Validation, Supervision, Formal analysis.

## Availability of data, code and other materials

All data analyzed in this review were extracted from published articles and are presented in the main text, tables, and supplementary material. The data extraction forms and analysis code are available from the corresponding author on reasonable request.

## Human and animal rights

This article does not contain any new studies with human or animal subjects performed by any of the authors. This systematic review and meta-analysis was conducted exclusively using data extracted from previously published studies. Each of the five included randomized controlled trials reported approval by the relevant institutional ethics committee and conduct in accordance with the ethical standards of the responsible committee on human experimentation (institutional and national) and with the 1964 Declaration of Helsinki and its later amendments. No animal studies were included in this review.

## Support/funding

This review received no specific grant from any funding agency in the public, commercial, or not-for-profit sectors. The authors received no financial or non-financial support for the conduct of this review. The funders had no role in the study design, data collection, data analysis, manuscript preparation, or decision to publish.

## Declaration of competing interest

The authors declare that they have no known competing financial interests or personal relationships that could have appeared to influence the work reported in this paper.
