# Supplementary material for: Effectiveness of platelet-rich fibrin (PRF) as adjunctive therapy in alveolar bone grafting: A systematic review and meta-analysis
Source: J Oral Biol Craniofac Res. 2026 Nov 3;16(6):101568. doi: 10.1016/j.jobcr.2026.101568 (PMC13756067; doi:10.1016/j.jobcr.2026.101568)
Supplement: Supplementary file 1 [file mmc1.docx]

**Supplementary Material**

##### Supplementary Table1. Keywords used to search each database

| **Database** | **Keyword** | **Total** |
| --- | --- | --- |
| **Pubmed**  **July 11 2026** | ("Alveolar Bone Grafting"[MeSH Terms] OR "Alveolar Bone Defects" OR "Alveolar Cleft" OR "Cleft Alveolus") AND ("Platelet-Rich Fibrin"[Mesh Terms] OR “PRF OR “leukocyte platelet-rich fibrin” OR L-PRF” OR “advanced platelet-rich fibrin” OR “A-PRF” OR “i-PRF”) AND ("Bone Regeneration"[MeshTerms] OR "Osteogenesis" OR "Bone Healing" OR "Bone Formation" OR "Bone Density" OR "Bone Volume" OR “Newly Formed Bone” OR "Graft Success") | **846** |
| **Cochrane**  **July 11 2026** | (alveolar bone graft):ti,ab,kw OR (alveolar bone grafting):ti,ab,kw OR (secondary alveolar bone graft):ti,ab,kw OR (alveolar cleft):ti,ab,kw OR (alveolar cleft reconstruction):ti,ab,kw AND (platelet-rich fibrin):ti,ab,kw OR (PRF):ti,ab,kw OR (leukocyte platelet-rich fibrin):ti,ab,kw OR (L-PRF):ti,ab,kw OR (advanced platelet-rich fibrin):ti,ab,kw  AND (bone regeneration):ti,ab,kw (Osteogenesis):ti,ab,kw OR (bone formation):ti,ab,kw OR (bone healing):ti,ab,kw OR (bone density):ti,ab,kw OR (Bone Volume):ti,ab.kw OR (Newly Formed Bone):ti,ab,kw OR (graft success):ti,ab,kw | **161** |
| **Embase**  **July 11 2026** | ('alveolar bone graft'/exp OR 'alveolar bone grafting':ti,ab OR 'secondary alveolar bone grafting':ti,ab) AND ('cleft lip palate'/exp OR 'alveolar cleft':ti,ab OR 'cleft palate':ti,ab) AND ('platelet rich fibrin'/exp OR 'platelet-rich fibrin':ti,ab OR PRF:ti,ab) AND ('bone regeneration'/exp OR 'bone healing'/exp OR 'bone volume':ti,ab OR 'bone density':ti,ab) | **92** |
| **Hand Search** | - | **2** |
| **T**otal | | **1.101** |

##### Supplementary Table 2. Comparison of the present review with previous systematic reviews of platelet concentrates in secondary alveolar bone grafting

| **Review** | **Design; search date** | **Platelet concentrate** | **Included studies** | **Outcomes** | **Synthesis and certainty** |
| --- | --- | --- | --- | --- | --- |
| Thangarajah et al., 2024^5^ | SR; to October 2022 | PRF and PRP together | 4 RCTs | Bone height, density, volume | No meta-analysis; outcomes similar with and without PRF/PRP |
| Campolo et al., 2024^8^ | SR and MA; to July 2022 | PRP or PRF (pooled as autologous platelet derivatives) | 12 RCTs | Newly formed bone, height and width loss, density, function, complications | PRP and PRF pooled; very low certainty for newly formed bone (GRADE) |
| Siddiqui et al., 2024^7^ | SR and MA; to July 2023 | Autologous platelet concentrates | 7 (6 RCTs, 1 CCT) | Wound dehiscence | RR 0.33 (95% CI 0.16 to 0.71) for dehiscence; bone outcomes not assessed |
| Baghaei et al., 2025^1^ | SR and MA; to July 2024 | Not PRF-specific (autograft, substitutes, combinations) | 10 RCTs (186 patients) | Bone density, volume, height, formation rate | Grouped by graft strategy; PRF not analyzed as a separate comparison |
| Present review | SR and MA; to July 2026 | PRF only | 5 RCTs (126 patients) | Newly formed bone, graft height, graft volume, bone density | Separate PRF meta-analyses with HKSJ sensitivity analysis; very low certainty (GRADE) |

CCT, controlled clinical trial; HKSJ, Hartung–Knapp–Sidik–Jonkman; MA, meta-analysis; PRF, platelet-rich fibrin; PRP, platelet-rich plasma; RCT, randomized controlled trial; RR, risk ratio; SR, systematic review.
